# Muscle Mitochondrial ATP Synthesis and Glucose Transport/Phosphorylation in Type 2 Diabetes

**DOI:** 10.1371/journal.pmed.0040154

**Published:** 2007-05-01

**Authors:** Julia Szendroedi, Albrecht I Schmid, Marek Chmelik, Christian Toth, Attila Brehm, Martin Krssak, Peter Nowotny, Michael Wolzt, Werner Waldhausl, Michael Roden

**Affiliations:** 1 Department of Internal Medicine 3, University of Vienna, Vienna, Austria; 2 Karl-Landsteiner Institute of Endocrinology and Metabolism, Vienna, Austria; 3 High-Field Magnetic Resonance Centre of Excellence, Medical University of Vienna, Vienna, Austria; 4 First Medical Department, Hanusch Hospital, Vienna, Austria; Yale Medical School, United States of America

## Abstract

**Background:**

Muscular insulin resistance is frequently characterized by blunted increases in glucose-6-phosphate (G-6-P) reflecting impaired glucose transport/phosphorylation. These abnormalities likely relate to excessive intramyocellular lipids and mitochondrial dysfunction. We hypothesized that alterations in insulin action and mitochondrial function should be present even in nonobese patients with well-controlled type 2 diabetes mellitus (T2DM).

**Methods and Findings:**

We measured G-6-P, ATP synthetic flux (i.e., synthesis) and lipid contents of skeletal muscle with ^31^P/^1^H magnetic resonance spectroscopy in ten patients with T2DM and in two control groups: ten sex-, age-, and body mass-matched elderly people; and 11 younger healthy individuals. Although insulin sensitivity was lower in patients with T2DM, muscle lipid contents were comparable and hyperinsulinemia increased G-6-P by 50% (95% confidence interval [CI] 39%–99%) in all groups. Patients with diabetes had 27% lower fasting ATP synthetic flux compared to younger controls (*p* = 0.031). Insulin stimulation increased ATP synthetic flux only in controls (younger: 26%, 95% CI 13%–42%; older: 11%, 95% CI 2%–25%), but failed to increase even during hyperglycemic hyperinsulinemia in patients with T2DM. Fasting free fatty acids and waist-to-hip ratios explained 44% of basal ATP synthetic flux. Insulin sensitivity explained 30% of insulin-stimulated ATP synthetic flux.

**Conclusions:**

Patients with well-controlled T2DM feature slightly lower flux through muscle ATP synthesis, which occurs independently of glucose transport /phosphorylation and lipid deposition but is determined by lipid availability and insulin sensitivity. Furthermore, the reduction in insulin-stimulated glucose disposal despite normal glucose transport/phosphorylation suggests further abnormalities mainly in glycogen synthesis in these patients.

## Abstract 2

Michael Roden and colleagues report that even patients with well-controlled insulin-resistant type 2 diabetes have altered mitochondrial function.

## Editors' Summary

**Background.:**

Diabetes mellitus is an increasingly common chronic disease characterized by high blood sugar (glucose) levels. In normal individuals, blood sugar levels are maintained by the hormone insulin. Insulin is released by the pancreas when blood glucose levels rise after eating (glucose is produced by the digestion of food) and “instructs” insulin-responsive muscle and fat cells to take up glucose from the bloodstream. The cells then use glucose as a fuel or convert it into glycogen, a storage form of glucose. In type 2 diabetes, the commonest type of diabetes, the muscle and fat cells become nonresponsive to insulin (a condition called insulin resistance) and consequently blood glucose levels rise. Over time, this hyperglycemia increases the risk of heart attacks, kidney failure, and other life-threatening complications.

**Why Was This Study Done?:**

Insulin resistance is often an early sign of type 2 diabetes, sometimes predating its development by many years, so understanding its causes might provide clues about how to stop the global diabetes epidemic. One theory is that mitochondria—cellular structures that produce the energy (in the form of a molecule called ATP) needed to keep cells functioning—do not work properly in people with insulin resistance. Mitochondria change (metabolize) fatty acids into energy, and recent studies have revealed that fat accumulation caused by poorly regulated fatty acid metabolism blocks insulin signaling, thus causing insulin resistance. Other studies using magnetic resonance spectroscopy (MRS) to study mitochondrial function noninvasively in human muscle indicate that mitochondria are dysfunctional in people with insulin resistance by showing that ATP synthesis is impaired in such individuals. In this study, the researchers have examined both baseline and insulin-stimulated mitochondrial function in nonobese patients with well-controlled type 2 diabetes and in normal controls to discover more about the relationship between mitochondrial dysfunction and insulin resistance.

**What Did the Researchers Do and Find?:**

The researchers determined the insulin sensitivity of people with type 2 diabetes and two sets of people (the “controls”) who did not have diabetes: one in which the volunteers were age-matched to the people with diabetes, and the other containing younger individuals (insulin resistance increases with age). To study insulin sensitivity in all three groups, the researchers used a “hyperinsulinemic–euglycemic clamp.” For this, after an overnight fast, the participants' insulin levels were kept high with a continuous insulin infusion while blood glucose levels were kept normal using a variable glucose infusion. In this situation, the glucose infusion rate equals glucose uptake by the body and therefore measures tissue sensitivity to insulin. Before and during the clamp, the researchers used MRS to measure glucose-6-phosphate (an indicator of how effectively glucose is taken into cells and phosphorylated), ATP synthesis, and the fat content of the participants' muscle cells. Insulin sensitivity was lower in the patients with diabetes than in the controls, but muscle lipid content was comparable and hyperinsulinemia increased glucose-6-phosphate levels similarly in all the groups. Patients with diabetes and the older controls had lower fasting ATP synthesis rates than the young controls and, whereas insulin stimulation increased ATP synthesis in all the controls, it had no effect in the patients with diabetes. In addition, fasting blood fatty acid levels were inversely related to basal ATP synthesis, whereas insulin sensitivity was directly related to insulin-stimulated ATP synthesis.

**What Do These Findings Mean?:**

These findings indicate that the impairment of muscle mitochondrial ATP synthesis in fasting conditions and after insulin stimulation in people with diabetes is not due to impaired glucose transport/phosphorylation or fat deposition in the muscles. Instead, it seems to be determined by lipid availability and insulin sensitivity. These results add to the evidence suggesting that mitochondrial function is disrupted in type 2 diabetes and in insulin resistance, but also suggest that there may be abnormalities in glycogen synthesis. More work is needed to determine the exact nature of these abnormalities and to discover whether they can be modulated to prevent the development of insulin resistance and type 2 diabetes. For now, though, these findings re-emphasize the need for people with type 2 diabetes or insulin resistance to reduce their food intake to compensate for the reduced energy needs of their muscles and to exercise to increase the ATP-generating capacity of their muscles. Both lifestyle changes could improve their overall health and life expectancy.

**Additional Information.:**

Please access these Web sites via the online version of this summary at http://dx.doi.org/10.1371/journal.pmed.0040154.

- The MedlinePlus encyclopedia has pages on diabetes
- The US National Institute of Diabetes and Digestive and Kidney Diseases provides information for patients on diabetes and insulin resistance
- The US Centers for Disease Control and Prevention has information on diabetes for patients and professionals
- American Diabetes Association provides information for patients on diabetes and insulin resistance
- Diabetes UK has information for patients and professionals on diabetes

## Introduction

Skeletal muscle insulin resistance is characteristic in the elderly as well as in persons at increased risk of type 2 diabetes mellitus (T2DM) and those with overt T2DM. In these groups, the content of intramyocellular lipids (IMCL) is frequently increased and related to insulin resistance [1,2]. However, this relationship disappears during exercise training, which increases both IMCL and insulin sensitivity in parallel [3,4]. Rather than IMCL, intracellular metabolites of free fatty acids (FFAs), such as long-chain fatty acyl coenzyme A and diacylglycerol, inhibit insulin action by stimulating phosphorylation of serine residues of insulin receptor substrate-1 (IRS-1) [5]. This suggests that IMCL do not directly contribute to insulin resistance but accumulate as a consequence of increased lipid availability from augmented lipolysis and excess dietary fat supply and/or of impaired mitochondrial lipid oxidation [6].

In severe obesity and moderately controlled T2DM, insulin resistance has been linked to abnormal mitochondrial function of skeletal muscle as assessed by in vitro and ex vivo examination in biopsies [7–9]. Application of magnetic resonance spectroscopy (MRS) made it possible to noninvasively examine myocellular mitochondrial function by measuring rates of ATP synthesis (“synthetic flux”) in humans [10]. Insulin resistant elderly people and first-degree relatives of patients with T2DM have impaired muscle ATP production and elevated IMCL [11,12]. These studies raised the questions of (i) whether lipid accumulation in skeletal muscle and/or insulin resistance promotes mitochondrial dysfunction and, if so, (ii) whether insulin stimulation can overcome such alterations. In this context, we recently showed that short-term elevation of plasma FFAs profoundly inhibits insulin-stimulated glucose transport/phosphorylation and mitochondrial function, reflected by diminished increase in intramyocellular glucose-6-phosphate (G-6-P) and ATP synthesis, independently of ectopic muscle fat contents [13].

Thus, this study examines relationships between basal and insulin-stimulated mitochondrial function, glucose disposal, and ectopic lipids in patients with T2DM and healthy controls. We employed multinuclear MRS to quantify ATP synthetic flux, intracellular phosphorus metabolites (G-6-P and inorganic phosphate [P_i_]), IMCL, and hepatocellular lipids (HCL) in combination with the stable isotope dilution technique to assess glucose fluxes during normoglycemic– and hyperglycemic–hyperinsulinemic clamps.

## Methods

### Volunteers

Thirty-one volunteers were included in this study: (i) patients with metabolically well-controlled T2DM, (ii) sex-, age-, body mass index (BMI)-matched older volunteers without diabetes (CONo) (age: 95% CI 52–63 y), and (iii) younger individuals without diabetes (CONy) (age: 95% CI 25–28 y). Their anthropometrical and laboratory characteristics are summarized in Table 1. All participants were recruited by means of public advertisement and underwent a complete medical history, clinical examination, and lab tests to exclude cardiovascular, hepatic, renal, and thyroid diseases. Patients with diabetes on insulin treatment or presenting with islet cell antibodies and diabetes-related complications were excluded. Five patients with T2DM had a confirmed family history of diabetes. The individuals in the control groups (CON) had neither a family history of diabetes nor were taking any medication on a regular basis. Normal glucose metabolism was confirmed by a standard oral 75 g glucose tolerance test. None of the participants performed intense exercise on a regular basis. Physical activity was assessed with a self-administered questionnaire of habitual physical activity [14]. This questionnaire discriminates between three features of physical activity: (i) physical activity at work (ii) sport during leisure time and (iii) physical activity during leisure time excluding sports. Average physical activity was calculated as the mean value across these features. The protocol was approved by the local institutional ethics board, and informed consent was obtained from each volunteer after explanation of the purpose, nature, and potential complications of the study.

**Table 1:**
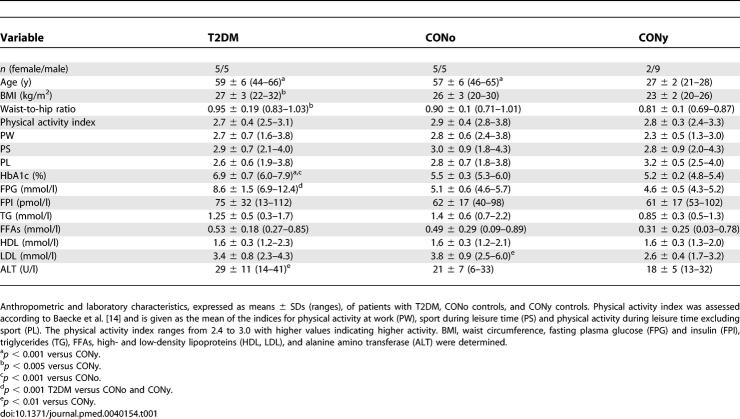
Clinical Characteristics of Volunteers

### Experimental Protocol

All participants were on an isocaloric diet, refrained from any physical exercise for 3 d prior to the study day, and fasted for 12 h before the start of the experiment. Glucose-lowering medication was withdrawn for at least 3 d before the experiment. All studies were started at 6:30 a.m. with the insertion of catheters (Vasofix; Braun, http://www.bbraun.com/index.cfm) in the antecubital veins of both arms for blood sampling and infusions. A primed-continuous infusion for 5 min ([3.6 mg fasting glucose (mg/dl)/90 (mg/dl)]/[min × kg body weight]); and for 480 min (0.036 mg/[min × kg body weight]) of D-[6,6-^2^H_2_]glucose (98% enriched; Cambridge Isotope Laboratories, http://www.isotope.com/cil/index.cfm) was performed until the end of the clamp test to determine endogenous glucose production (EGP). At 7:00 a.m., all participants were transferred to the Magnetic Resonance Unit to measure IMCL, G-6-P, and flux through ATP synthase in calf muscle using ^1^H/^31^P MRS. After basal measurements, the clamp test was started at 10:00 a.m. to create conditions of normoglycemia (approximately 5.5 mmol/l glucose) and hyperinsulinemia (approximately 500 pmol/l insulin). Insulin (Actrapid; Novo Nordisk, http://www.novonordisk.com/) was administered as a primed-continuous infusion (40 mU/[m^2^ body surface area × min]) from 0 to 360 min. A 20% dextrose infusion labeled with D-[6,6-^2^H_2_]glucose (2% enriched) according to the hot glucose infusion (hot GINF) protocol was periodically adjusted to maintain normoglycemia [15]. Whole body insulin sensitivity was assessed from whole-body glucose disposal (M) during the last 30 min of the clamp. ^31^P MRS measurements (G-6-P, P_i_, ATP synthesis) were repeated between 120 min and 240 min. Four of the patients with T2DM (three males and one female, mean values ± SD: age, 62 ± 2 y, BMI, 27.5 ± 3 kg/m^2^; M, 4.8 ± 2.5 mg/(min × kg body weight) underwent an additional hyperglycemic (approximately 9.5 mmol/l glucose) –hyperinsulinemic (approximately 500 pmol/l insulin) clamp test on a separate day to examine the combined effect of insulin and glucose on G-6-P and ATP synthesis.

### Magnetic Resonance Spectroscopy

Measurements were performed on participants lying supine inside a 3-T spectrometer (Bruker, http://www.bruker.com/) using a 10 cm circular double resonant surface coil for ^1^H and ^31^P measurements, positioned approximately 2 cm into the medial head of the right gastrocnemius muscle as described previously [13].

### ^31^P MRS

^31^P spectra were acquired at baseline (−100 to −20 min) and during the last 80 min of the clamp. Rates of basal and insulin-stimulated skeletal muscle ATP synthetic flux (bfATP and ifATP, respectively) were assessed with ^31^P MRS using the saturation transfer experiment applied to the exchange between P_i_ and ATP [11,13,16]. Intramyocellular concentrations of G-6-P (μmol/l muscle) and P_i_ were measured from the ratio of the integrated respective peak intensities and β-ATP resonance intensity in spectra without inversion and saturation assuming a constant ATP concentration of 5.5 mmol/l muscle [2].

### ^1^H MRS

IMCL in soleus muscle was determined by ^1^H MRS as described [1]. The cubic volume of interest within the muscle (1.73 cm^3^) and the magnetic field was shimmed on the localized water signal (line width, 2–15 Hz). On a separate day and after a 12-h fast, localized ^1^H MRS of the liver was acquired to measure HCL as described [17].

### Analytical Procedures

Plasma glucose was measured by the glucose oxidase method (Glucose analyzer II, Beckman Coulter, http://www.beckmancoulter.com/). Plasma FFAs were assayed with a microfluorimetric method (Wako Chem USA, http://www.wakousa.com/). In vitro lipolysis was prevented by collecting blood into orlistat-containing vials. Plasma triglycerides were measured by a peroxidase-coupled colorimetric assay (Roche, http://www.roche.com/home.html). Plasma concentrations of insulin, C-peptide and glucagon were determined by double antibody radioimmunoassay [15]. Plasma lactate concentrations were measured enzymatically (Roche) [17].

### Calculations and Statistics

Basal rates of glucose appearance (*R*_a_) were calculated by dividing the tracer D-[6,6-^2^H_2_] glucose infusion rate times tracer enrichment by the percent of tracer enrichment in plasma and subtracting the tracer infusion rate [18]. *R*_a_ was calculated using Steele's nonsteady-state equations [19]. EGP was calculated from the difference between *R*_a_ and mean glucose infusion rates. Suppression of EGP (ΔEGP) by insulin is given as the percent change of EGP at 240 min from fasting. Suppression of plasma FFAs by insulin was calculated analogous to EGP.

All statistical analyses were performed using SPSS 6.0 software (SPSS, http://www.spss.com). Data are presented as means ± standard deviations (SDs) throughout the text and in the figures. Statistical comparisons between study groups were performed using ANOVA and repeated measurements ANOVA with Tukey post hoc testing when appropriate. Within-group differences were determined using two-tailed Student's t tests. Non-parametric correlations are Spearman correlations (*R, P*). Multiple linear regression analysis was performed for the dependent variable, bfATP, including the following basal metabolic parameters: C-peptide, alanine aminotransferase, HbA1c, fasting plasma glucose, basal FFAs, basal EGP, M, HCL, IMCL, basal G-6-P, activity index, BMI, waist-to-hip ratio, and age. Regression analysis for the dependent variable, ifATP, was done using the following independent variables: ΔEGP, suppression of FFAs and increase in G-6-P during clamp, M, HCL, IMCL, activity index, BMI, waist-to-hip ratio, and age. Differences were considered significant at the 5% level.

## Results

### Baseline Characteristics

Volunteers in the T2DM group were nonobese, normolipidemic, and metabolically well-controlled with stable HbA1c values over the course of the last two years (Table 1). Basal EGP was not different from controls (T2DM, 1.8 ± 0.4 mg/[kg body weight × min]; CONo, 1.9 ± 0.3 mg/[kg body weight × min]; CONy, 1.7 ± 0.6 mg/[kg body weight × min]; *p* = 0.858).

### Whole Body Metabolism During Normoglycemic-Hyperinsulinemic Clamps

**Figure 1:**
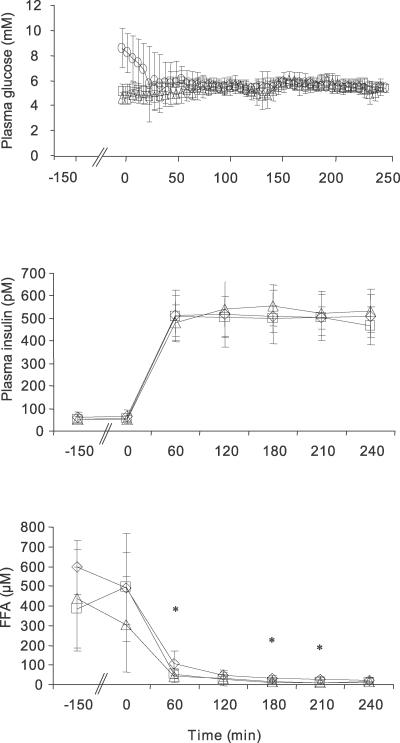
Plasma Glucose, Insulin and FFAs Time course of plasma glucose (top), insulin (middle), and FFAs (bottom) concentrations during euglycemic (approximately 5.5 mM glucose) –hyperinsulinemic (approximately 500 pM insulin) clamps in patients with T2DM (*n* = 10) (diamonds), age- and BMI-matched controls (CONo; *n* = 10) (squares), and young healthy controls (CONy; *n* = 11) (triangles). All units expressed as means ± SD. * *p* < 0.05 T2DM versus controls.

Fasting plasma glucose was higher in patients with T2DM but normalized during the first 30 min of the clamps (Figure 1). In patients with T2DM, fasting plasma C-peptide was 61% higher than in both control groups (T2DM, 1.2 ± 0.3 nmol/l; CONo, 0.8 ± 0.1 nmol/l; CONy, 0.7 ± 0.1 nmol/l; *p* < 0.001 versus T2DM). Nevertheless, fasting plasma insulin was comparable in all groups (Table 1). During the clamps, mean concentrations of glucose and insulin were 5.5 ± 0.5 mmol/l and 514 ± 96 pmol/l, respectively, and did not differ between the groups (Figure 1). Fasting plasma lactate was lower (*p* = 0.005) in the CONy group than in patients with T2DM (T2DM, 1.26 ± 0.41 mmol/l; CONo, 1.01 ± 0.25 mmol/l; CONy, 0.80 ± 0.25 mmol/l). During the clamp, lactate concentrations remained unchanged in patients with T2DM, gradually increased in the CONo participants (*p* = 0.075) and increased in the CONy participants by 56% (*p* = 0.017) (T2DM, 1.30 ± 0.77 mmol/l; CONo, 1.29 ± 0.39 mmol/l; CONy, 1.24 ± 0.11 mmol/l). Insulin-mediated suppression of FFAs was comparable in all groups (T2DM, 92 *±* 6%; CONo, 95 *±* 4%; CONy, 90 *±* 8%), although patients with T2DM had slightly higher plasma FFAs at 60, 180, and 210 min; *p* < 0.05 (Figure 1).

**Figure 2:**
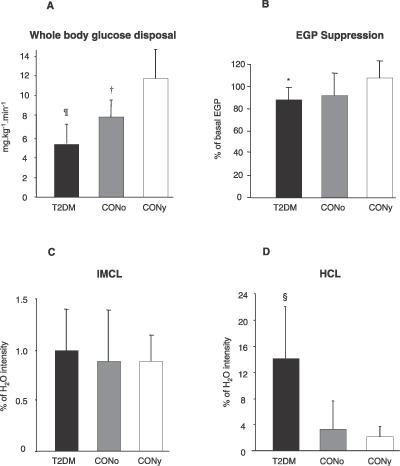
Glucose Metabolism and Intracellular Lipids of Skeletal Muscle and Liver Whole-body glucose disposal (A) and ΔEGP (B) during euglycemic–hyperinsulinemic clamp (*n* = 31). IMCL in skeletal muscle (*n* = 31) (C) and liver (*n* = 29) (HCL) (D). Patients with T2DM (black columns), CONo (grey columns), and CONy (white columns). All results are means *±* SD. ¶ *p* < 0.001 T2DM versus CONy; † *p* < 0.01 CONo versus T2DM and CONy; * *p* < 0.05 T2DM versus CONy; § *p* < 0.001 T2DM versus controls.

Insulin-stimulated whole body glucose disposal (M) was 126% and 54% higher in CONy and CONo participants than in patients with T2DM, respectively (*p* < 0.001, *p* = 0.010) (Figure 2). Insulin-stimulated suppression of EGP was more pronounced in CONy participants than in patients with T2DM (ΔEGP: T2DM, 88% *±* 8%; CONo, 91% *±* 21%; CONy, 107% *±* 14%; *p* < 0.032 T2DM versus CONy).

### Intracellular Metabolites

IMCL did not differ between the groups (T2DM, 1.0% *±* 0.5%; CONo, 0.9% *±* 0.5%; CONy, 0.9% *±* 0.3%; *p* = 0.808) (Figure 2), whereas HCL were 4- to 7-fold higher in patients with T2DM (T2DM, 14.1% *±* 8.3%; CONo, 3.3% *±* 4.4%; CONy, 2.2% *±* 1.8%; *p* < 0.001 versus T2DM).

Intramyocellular G-6-P concentrations were similar during fasting (0.15 *±* 0.057 mmol/l muscle) and increased by 50% during the clamp in all groups (0.22 *±* 0.071 mmol/l muscle: T2DM, *p* = 0.044 versus baseline; CONo, *p* = 0.009 versus baseline; CONy, *p* = 0.002 versus baseline). Likewise, intramyocellular P_i_ concentrations were comparable at baseline (2.9 *±* 0.4 mmol/l muscle) and increased 17% during the clamp in all groups (3.4 *±* 0.4 mmol/l muscle: T2DM, *p* = 0.005 versus baseline; CONo, *p* = 0.001 versus baseline; CONy, *p* = 0.004 versus baseline).

### Rates of Muscle ATP Synthesis

**Figure 3:**
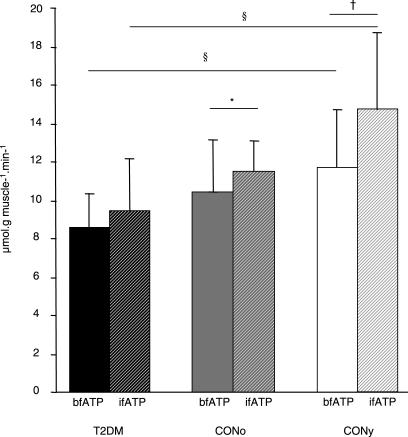
Rates of ATP Synthetic Flux Rates of ATP synthetic flux (means *±* SD) during fasting (bfATP, full columns) and during insulin stimulation (ifATP, hatched columns). Patients with T2DM (black columns), CONo controls (grey columns) and CONy controls (white columns) (*n* = 31). § *p* < 0.05 T2DM versus CONy; *, *p* < 0.05; †, *p* < 0.01.

**Figure 4:**
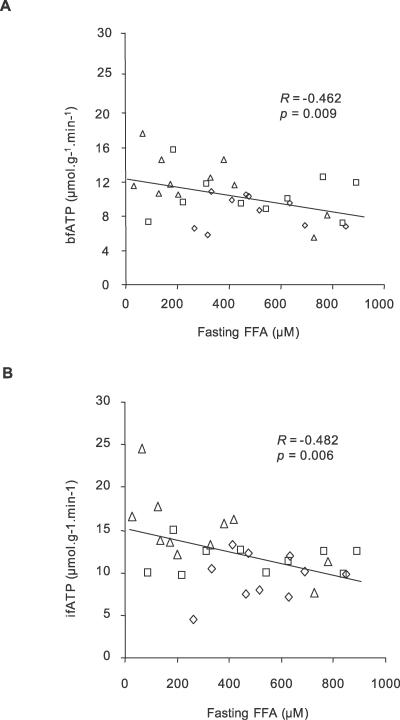
Associations between ATP Synthetic Flux and Metabolic Parameters Correlation analyses of rates of ATP synthetic flux (*n* = 31). (A) Relation between fasting plasma concentration of FFAs and bfATP across all groups. (B) Relation between fasting plasma FFAs and ifATP across all groups. Patients with T2DM (*n* = 10) (diamonds), age- and BMI-matched controls (CONo; *n* = 10) (squares), and young healthy controls (CONy; *n* = 11) (triangles).

bfATP was 27% lower in patients with T2DM than in CONy participants (T2DM, 8.6 *±* 1.9 μmol/[g muscle × min]; CONy, 11.8 *±* 3.3 μmol/[g muscle × min] *p* < 0.031) and did not differ from CONo participants (10.4 *±* 2.6 μmol/[g muscle × min]; *p* = 0.293 versus T2DM and *p* = 0.505 versus CONy) (Figure 3). ifATP rose by 26% and 11% in the CONy participants (14.8 *±* 4.3 μmol/[g muscle × min], *p* = 0.003 versus basal) and the CONo participants (11.5 *±* 1.7 μmol/[g muscle × min], *p* = 0.023 versus basal), but not in patients with T2DM (9.5 *±* 2.7 μmol/[g muscle × min], *p* = 0.256 versus basal). Significant associations between ATP synthesis rates and physiological variables are shown in Table 2 and Figure 4. Fasting plasma insulin levels neither related to bfATP, ifATP, nor to M. C-peptide related negatively to ifATP (*R* = −0.458, *p* = 0.010) and positively to M (*R* = 0.550, *p* = 0.001). Adjustment for M disrupted the association between ATP synthetic fluxes and C-peptide levels. Adjustment for sex, age, BMI, and waist-to-hip ratio did not disrupt the negative correlations of bfATP and ifATP with fasting FFAs (*R* = −0.533, *p* = 0.007; *R* = −0.408, *p* = 0.042) and of ifATP to HCL (*R* = −0.415, *p* = 0.044). bfATP correlated positively with mean physical activity (*R* = 0.945, *p* < 0.001) and negatively with BMI (*R* = −0.818, *p* = 0.004) only in patients with T2DM. Multiple linear regression analysis for the dependent variable bfATP including basal metabolic parameters (see Methods) identified basal plasma FFAs and waist-to-hip ratio as significant and independent predictors of bfATP (Table 3). In model 1, basal FFAs alone explained 28% of the variance of bfATP. In model 2, waist-to-hip ratio was included in addition to basal plasma FFAs, thereby explaining 44% of the variance of bfATP. Multiple regression analysis for the dependent variable ifATP identified insulin sensitivity (M) as a significant predictor and explained 30% of the variance of ifATP (Table 3).

**Table 2:**
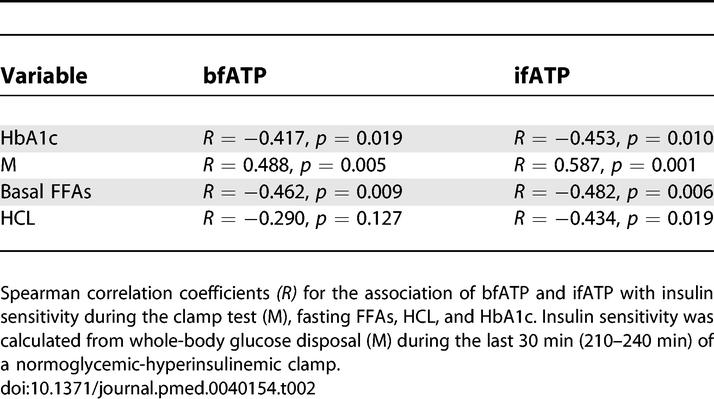
Associations between ATP Synthesis Rates and Physiological Variables

**Table 3:**
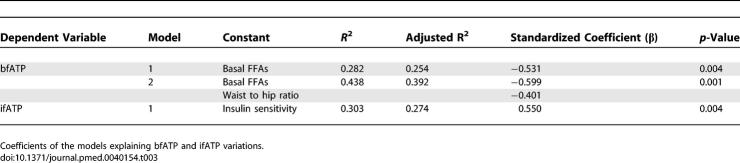
Multiple Regression Analysis for the Dependent Variables bfATP and ifATP

### Whole Body Metabolism During Hyperglycemic-Hyperinsulinemic Clamps

**Figure 5:**
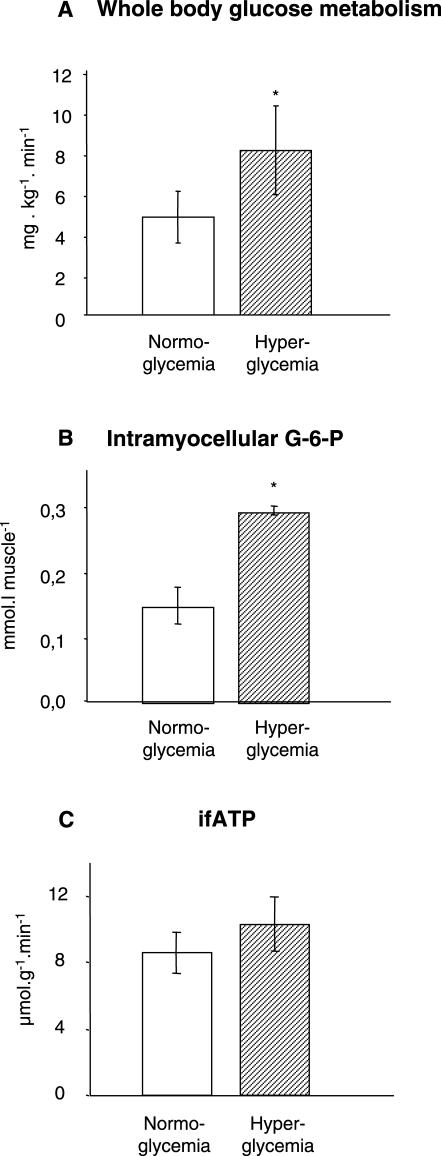
Glucose Metabolism and ATP Synthetic Flux during Hyperglycemic–Hyperinsulinemia in T2DM Whole body glucose disposal (A), concentration of intramyocellular G-6-P (B), and ifATP (C) during euglycemic (approximately 5.5 mM glucose)–hyperinsulinemic (approximately 500 pM insulin) (white columns) and hyperglycemic (approximately 9.5 mM glucose)–hyperinsulinemic (approximately 500 pM insulin) clamps (hatched columns) in patients with T2DM (*n* = 4). * *p* < 0.05.

The effect of combined glucose and insulin stimulation on ifATP was studied in four of the T2DM volunteers during hyperglycemic–hyperinsulinemic clamps (Figure 5). Mean plasma glucose almost doubled (9.6 *±* 0.8 mmol/l versus 5.5 *±* 0.3 mmol/l; *p* < 0.001) resulting in 70% higher M-values compared to normoglycemic hyperinsulinemia (8.1 *±* 4.4 mg/[kg body weight × min] versus 4.8 *±* 2.5 mg/[kg body weight × min]; *p* = 0.048). Intramyocellular G-6-P concentrations doubled compared to normoglycemic hyperinsulinemia (240 min: 0.29 *±* 0.01 mmol/l muscle versus 0.15 *±* 0.05 mmol/l muscle; *p* = 0.010). Mean bfATP, ifATP and change of fATP during insulin stimulation did not differ between normoglycemic and hyperglycemic clamps (bfATP: 9.5 *±* 2.2 μmol/[g muscle × min] versus 8.1 *±* 1.7 μmol/[g muscle × min], *p* = 0.506; ifATP: 10.3 *±* 2.4 μmol/[g muscle × min] versus 8.6 *±* 3.2 μmol/[g muscle × min], *p* = 0.156, *p* = 0.628 basal versus insulin stimulation during hyperglycemic–hyperinsulinemic clamp; change of fATP during insulin stimulation: 0.9 *±* 3.3 μmol/[g muscle × min] versus 0.03 *±* 1.8 μmol/[g muscle × min], *p* = 0.579).

## Discussion

Patients with well-controlled T2DM have impaired flux through muscle ATP synthesis occurring independently of glucose transport/phosphorylation and lipid deposition. Lipid availability primarily determines bfATP, whereas insulin sensitivity defines ifATP. Furthermore, the reduction in insulin-stimulated glucose disposal despite normal glucose transport/phosphorylation suggests further abnormalities, mainly in glycogen synthesis of these patients with T2DM.

We studied slightly overweight patients with well-controlled T2DM featuring a lower degree of whole-body insulin resistance compared with previous studies on mitochondrial function in insulin resistant states [9,20,21]. Of note, BMI, waist-to-hip ratio, fasting lipid, and insulin concentrations were comparable to an age-matched control group. These patients with overt T2DM exhibit somewhat reduced muscular ATP synthesis as assessed by noninvasive in vivo ^31^P MRS.

### Fasting Conditions

Both control groups featured similar bfATP, which was nearly doubled compared to a group of participants who were 15 years older, in a previous study [11]. Thus, aging-dependent alterations in mitochondrial number, morphology, and in vivo function do not seem to be present in our CONo group, but cannot be completely excluded, because we did not perform muscle biopsies in this study [5,11,22]. bfATP of the patients with T2DM was higher than in other insulin-resistant groups, but lower only compared to the CONy group [11,12,23]. This variation suggests that nondiabetic insulin-resistant groups, such as first-degree relatives of patients with T2DM, exhibit peculiar abnormalities in bfATP which can be confounded by secondary metabolic events in the overt diabetic state. Physical activity and body mass were the major determinants of bfATP only in the patients with T2DM, confirming the relationship between exercise training and mitochondrial function and further pointing to a specific susceptibility of mitochondrial function to lifestyle factors in patients with T2DM [24].

Fasting plasma FFAs and waist-to-hip ratio were identified as independent predictors of bfATP. FFAs could decrease ATP synthesis by protonophoric action on the inner mitochondrial membrane, activation of uncoupling protein-1 (UCP-1), and damage of mitochondrial proteins and DNA, resulting from formation of reactive oxygen species [25–28]. Lipid-induced dysfunction of mitochondria might also occur via down-regulation of nuclear genes involved in genes that are involved in oxidative phosphorylation (OXPHOS) and mitochondrial biogenesis [29]. Thus, environmental factors could serve as an epigenetic trigger affecting mitochondrial biogenesis in patients with T2DM [30]. The frequently observed inverse association between lipid accumulation in skeletal muscle and insulin sensitivity is influenced by various factors such as gender, ethnicity, genes, and total body fat content, and it depends on muscle fiber composition [1]. In the present study, IMCL did not differ between groups, suggesting that FFAs and their intracellular metabolites, rather than lipid deposition, are related to mitochondrial function.

Despite their good long-term metabolic control, the patients with T2DM were hyperglycemic during bfATP measurements due to withdrawal of glucose-lowering medication. Of note, transcription of OXPHOS genes can be normalized by near-normoglycemia in patients with poorly controlled T2DM [31]. Hyperglycemia also increases reactive oxygen species formation due to an imbalance between glucose phosphorylation by mitochondria-bound hexokinase and reactions decreasing G-6-P such as glycolysis, pentose phosphate shunt, and glycogen synthesis. The resulting rise in G-6-P would cause feedback inhibition of hexokinase, decrease the mitochondrial ADP:ATP ratio, and thereby disrupt ADP recycling [32,33]. As fasting intramyocellular G-6-P was not different between individuals with and without diabetes, increased plasma glucose cannot completely account for the reduction of bfATP in patients with T2DM.

### Insulin Stimulated Conditions

Although glucose disposal was markedly lower in patients with T2DM than in the controls during insulin stimulation, the 50% increase in G-6-P was surprisingly comparable between all three groups. Hyperinsulinemia of 515 pmol/l resulted in stimulation of glucose transport/phosphorylation in patients with T2DM similar to that in persons who do not have diabetes, and it rose even further during hyperglycemic hyperinsulinemia. The rise in G-6-P in the face of impaired glucose disposal could result from a defect of either glucose oxidation or glycogen synthesis relative to glucose transport via GLUT-4 and/or phosphorylation (hexokinase II), both of which lead to reduction of insulin-stimulated ATP synthesis.

Glucose transport was shown to be rate controlling for glucose disposal at lower plasma insulin concentrations and in obese patients with poorly controlled T2DM [34]. Reduced expression and/or flux through hexokinase II indicate that glucose phosphorylation can also be impaired in patients with T2DM [32]. One might speculate that the insulin-induced rise in G-6-P observed in our patients with T2DM could have reduced the activity of hexokinase II and lower the ADP:ATP ratio which in turn would slow down stimulation of ATP synthase flux [35]. However, both absolute concentrations and percentage increase of G-6-P were almost identical, rendering this possibility unlikely. Thus, the insulin-sensitive rise in G-6-P seems to depend critically on the degree of insulinemia and glycemia and probably also on long-term metabolic control. These mechanisms could explain why a 20-fold rise in plasma insulin concentrations during hyperglycemia completely normalizes the diminished insulin-stimulated increment in G-6-P of patients with poorly controlled T2DM [36]. On the other hand, lean insulin-resistant relatives of patients with T2DM exhibit a markedly reduced insulin-sensitive rise in G-6-P despite normoglycemia, highlighting the predominant role of inherited factors also for glucose transport/phosphorylation in this group [35].

Our patients with T2DM exhibited 27% lower rates of fasting ATP synthetic flux than the CONy participants, suggesting reduced aerobic capacity in T2DM. Reduction of oxidative capacity during insulin stimulation would diminish glucose oxidation—as reflected by impaired stimulation of ATP synthesis—and simultaneously cause reduction of glucose disposal. Plasma lactate was slightly higher during fasting but comparable during insulin stimulation. Carbohydrate oxidation was therefore likely unchanged, although abnormalities cannot be ruled out, because plasma lactate levels are also determined by extramuscular sources such as adipocytes [37].

In our patients with T2DM, impaired glucose disposal despite intact glucose transport/phosphorylation could further mirror a defect in insulin-stimulated nonoxidative glucose metabolism, i.e., muscle glycogen synthesis. In insulin-resistant nonobese relatives of patients with T2DM, exercise training unmasks an independent abnormality in muscle glycogen synthesis as shown by doubling of insulin-stimulated G-6-P without normalization of nonoxidative glucose metabolism [38]. One limitation of the present study resides in the lack of data on muscle glycogen synthesis, which could not be measured due to time restrictions in this protocol. Nevertheless, the available data suggest a distinct post-transport/phosphorylation defect, particularly in glycogen synthesis, in these patients with well-controlled T2DM [39–41]. Another limitation resides in the small sample size of this study, which affects the probability of detecting associations between parameters and differences between groups. Of note, some normality approximations do not necessarily hold true for small numbers.

In young healthy humans, insulin markedly stimulated ATP synthesis, which primarily results from augmented substrate availability, mainly glucose, and stimulation of respiratory chain enzyme activities within 1–3 h of hyperinsulinemia, and from increased mitochondrial protein expression after 6 h of prolonged hyperinsulinemia [7,13,23]. ATP synthesis rose only modestly in CONo participants (11%) but not in patients with T2DM. Despite the known age-related mitochondrial abnormalities, in vivo mitochondrial function can be maintained in humans up to 70 years of age depending on physical activity and body mass [42,43]. Likewise, insulin sensitivity can be unimpaired but also reduced upon correction for body mass in the elderly [11,44–46]. In the present study, adjustment for BMI and waist-to-hip ratio did not affect the association between age and insulin sensitivity (*R* = −0.634, *p* < 0.001). Moreover, the lower increase in ifATP in our elderly participants is in line with the previously observed simultaneous occurrence of decreased basal ATP synthesis and decreased insulin sensitivity, and supports the contention that age-dependent changes in ifATP relate to those of insulin sensitivity [11].

The participants with T2DM, to which the controls were matched for age and BMI, failed to exhibit an increase in ifATP, similar to recent findings in young nonobese insulin-resistant offspring of patients with T2DM. Thus, it can be hypothesized that insulin resistance is responsible for impaired ATP synthesis during insulin stimulation, which is supported by the multivariate analyses across all groups in the present study. The nonobese patients with T2DM featured defective ifATP despite normal G-6-P stimulation, suggesting an abnormality independent of glucose transport that does not disappear by matching glucose disposal to that of older controls and resulting in doubling of G-6-P by hyperglycemic hyperinsulinemia.

Intramuscular P_i_ concentrations increased by 17%, in agreement with previous studies [47]. The finding of similar basal and insulin-stimulated P_i_ in all groups seems to exclude abnormal P_i_ transport as the explanation for the ifATP variability in patients with T2DM. In contrast, lean insulin-resistant relatives of patients with T2DM exhibit markedly decreased insulin-stimulated P_i_ transport [23]. The difference between this group and our patients with overt T2DM could result from specific inherited mitochondrial abnormalities in the relatives or secondary metabolic changes obscuring the effect on P_i_ transport in the patients with T2DM. Alternatively, increased lipid availability could explain lower ifATP by interference with insulin signaling, which is in line with FFA-dependent ifATP inhibition [13]. Of note, plasma levels of FFAs during fasting was found to be tightly related to both bfATP and ifATP in the present study. Finally, ifATP correlated with HCL and elevation of HCL was identified as an early abnormality in patients with well-controlled T2DM, supporting the concept that ectopic fat in the liver rather than in muscle relates to whole body insulin sensitivity [48].

Different variants of insulin resistance can be found in humans. Insulin-resistant lean normoglycemic relatives of patients with T2DM present with IMCL accumulation along with clearly impaired insulin-stimulated P_i_ transport, mitochondrial ATP synthesis, and glucose transport and/or phosphorylation. In contrast, insulin-resistant nonobese near-normoglycemic patients with T2DM exhibit only slightly reduced ATP synthesis but have completely normal IMCL, P_i_ transport, and G-6-P increase. The difference could be explained by genes and secondary metabolic alterations such as glucolipotoxicity affecting the patients with T2DM. However, comparable fasting lipid concentrations and body mass in patients with T2DM and the age-matched control group render the latter possibility unlikely. To which extent accumulation of ectopic lipids (particularly HCL, which is seen in both offspring of and patients with overt T2DM) serve as common mediators or indicators of insulin resistance regardless of muscular abnormalities requires further investigation.

In conclusion, (i) patients with well-controlled insulin resistant T2DM have slightly lower ATP synthesis independent of glucose transport/phosphorylation and lipid deposition in muscle, (ii) lipid availability primarily determines bfATP, whereas (iii) insulin sensitivity defines ifATP, and (iv) reduction in insulin-stimulated glucose disposal suggests further abnormalities, mainly in glycogen synthesis of these patients with T2DM.

This study underlines the strong relationship between insulin sensitivity and function of mitochondria, the cells' power plants. Even a small degree of overweight and physical inactivity is associated with reduction in mitochondrial function, confirming the importance of lifestyle for development and prevention of insulin resistance and T2DM. Further research is needed, however, to delineate whether abnormalities in mitochondrial number and/or function are the cause or consequence of T2DM and to address the mitochondria as a target for novel therapeutic regimens.

## Abbreviations

bfATP: basal ATP synthetic flux
BMI: body mass index
CI: confidence interval
CONo: age, sex, and BMI-matched control volunteer(s)
CONy: young healthy control volunteer(s)
EGP: endogenous glucose production
ΔEGP: suppression of EGP
FFA: free fatty acid
G-6-P: glucose-6-phosphate
HCL: hepatocellular lipids
ifATP: insulin-stimulated ATP synthetic flux
IMCL: intramyocellular lipids
M: insulin-stimulated whole-body glucose disposal
MRS: magnetic resonance spectroscopy
Pi: inorganic phosphate
SD: standard deviation
T2DM: type 2 diabetes mellitus
